# Converting Food into Blood

**Published:** 1884-03

**Authors:** 


The process of converting food into blood is as follows:

After entering the stomach, it is converted into a sweetish whitish fluid in about two hours, when it is gradually passed out of the stomach along the intestines down to the vent of the system, receiving as it passes out of the stomach, drop by drop, the bile from the liver. In about four hours after eating an ordinary meal the stomach is empty, and in another hour or two we begin to get hungry. Opening into the stomach and all along the intestines, there are multitudes of open-mouthed tubes whose office it is to absorb, or withdraw what is real nutriment, frcm the passing mass of food, its essence. Some of it is ready to be withdrawn while in the stomach, other portions only become ready at various points along the intestinal passages, some only at the end. Thus it is that some elements of food are not converted into nutriment until long after having passed out of the stomach. These diminutive tubes convey their contents towards the great central tube of the system, just as the various springs, rivulets, creeks, &c., of any of our great rivers, flow together until all are united in one magnificent stream, which itself is finally emptied into the boundless sea. The heart is the great receiving sea of the myriads of nutriment-bearing channels of the human system. This nutrimental material enters the heart at the same time that another great river pours its contents into it; that river of blood which started from the heart a very few minutes before, and having washed out the body, delivers the defiled mass into the heart again to be renovated, refined, vivified. So that at any moment, the right side of the heart is industriously receiving two different kinds of fluid, the washings out of the body, and the imperfect nutrient material for blood, just as the Mississippi and Missouri pour very different waters together at their uniting point, soon mingling, however, into one homogeneous stream. The impure blood and the nutrient material soon coalesce, commingle and enter the lungs for purification, thoroughly mixed together. There meeting with the air, the nutrient fluid is in an instant converted into pure blood, and in the same instant of time, are the washings of the system converted into blood equally pure, by having had all its impurities abstracted at a breath. Thus we see, that in reality, our food does not become living, actual blood, until it has entered the lungs and been exposed to the life-giving influences of the air therein; hence we see that if air has not its life, that is its purity, it is utterly impossible for the food we have eaten to receive that finishing stroke which makes it real, perfect blood. And if not perfect, the system is imperfectly fed, and debility and disease are inevitable.

After the air in the lungs has given the finishing stroke, which makes pure and perfect blood out of the heterogeneous mass before described, it is sent back to the great receiving reservoir of the system, the left side of the heart, and is sent by thousands of *distributing* pipes, or blood vessels, to every fibre of the human frame, to be made into flesh, and bone, and joint, and ligament, wherever renovation is needed. And how minutely grand the process. The instant the air meets the impure and imperfect fluid mass in the lungs, it is converted into life, as instantaneous as chrystalization, as quick as the very lightning. This life consists in forming a little boat or cell, like a Nautilus on the sea; in this boat is an atom of life-giving life, which is freighted along the current of the blood, until it arrives at its destined port; the instant of its striking, the vessel is broken, the living atom, as instantaneously as the needle to the armature, bounds to its new home and is a part of the living man, in its turn to die and be washed away to make place for others. How wonderful is our life! How grandly mysterious, and how beautifully wise, is He who made it.

